# Reducing diagnostic errors in emergency department with the help of radiographers

**DOI:** 10.1002/jmrs.351

**Published:** 2019-08-27

**Authors:** Madhukar Thakkalpalli

**Affiliations:** ^1^ Emergency Department Logan Hospital Meadowbrook Queensland Australia

## Abstract

A radiographer has the unique advantage of directly seeing the patient and presenting injury at the time of imaging. Extending this skill set to further evaluate the image acquired and document the findings may be helpful in reducing diagnostic errors in emergency department.

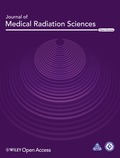

Since the invention of X‐rays, medical imaging has played a prodigious role in the field of medicine, with it being an important and at times the only diagnostic tool amongst an array of investigative methods available to a healthcare practitioner. Emergency departments (ED) form the frontline defence for a healthcare system, and with increasing activity seen throughout Australian departments,^1^ providing safe and efficient care to patients can be challenging. In 2017–2018 injury, poisoning and certain other consequences of external causes accounted for 25% of presentations to ED in Australia^1^ of which injury is likely to be the major player. In contemporary clinical practice, the majority of patients presenting at Australian EDs are initially assessed by a junior doctor along with interpretation of necessary laboratory and radiological tests. These initial interpretations are frequently discussed with their senior counterparts who include both ED registrars and ED senior medical officers/consultants. Immediate reporting of radiographic images by radiologists can significantly reduce interpretative errors and decrease patient recalls; however, this is not standard practice in ED worldwide.^2^

Due to the constant rotation of junior doctors in and out of the ED, coupled with the spectrum of skills that junior doctors are required to develop, the consultant group in my ED are always considering new processes/initiatives that assist in mitigating error. One such initiative is radiographer preliminary image evaluation (PIE). The introduction of a PIE system in 2016 into my ED has seen an extra layer of safety implemented for patients whose radiographic images are interpreted by referring healthcare practitioners such as medical doctors, nurse practitioners and physiotherapists. Healthcare practitioners are advised to initially review the radiographic images and make an assessment before checking their assessment against the radiographer PIE. This workflow assists referrers in developing their image interpretation skills by having an immediate second opinion.

A radiographer has the unique advantage of directly seeing the patient (and presenting injury) at the time of imaging. This scenario along with their knowledge and expertise of radiography promotes the acquisition of high‐quality X‐ray imaging. This also provides the radiographer with an extra edge at interpreting the radiographic images when compared to the referring practitioner and the reporting radiologist. Radiographers in a public healthcare system may form a stable workforce when compared to junior doctors who tend to rotate between multiple specialities, and training a radiographer would be more cost‐effective and may result in consistent image interpretation.

In reference to the study by Brown et al.^3^ published in the current edition of this journal, this study reported a sensitivity of 71.1% which could result from a combination of factors that may include a lack of specific training. Additionally, the study participants also included junior radiographers, and this highlights the fact that there is still room for improvement in radiographer PIE performance which can likely be addressed by dedicated training of the radiographers or utilising more senior radiographers for leading the PIE system.

Following review of the findings of this audit, an additional process was employed to reduce errors that may have occurred from the radiographer's incorrect PIE. This process involves the reporting radiologist flagging examinations where a discrepancy exists between the radiographer's PIE and the radiologist's report. These discrepancies are reviewed by a senior radiographer who compares the radiologist report to the ED diagnosis. If a true discrepancy exists, the senior radiographer notifies an ED consultant and requests the case to be reviewed. I believe this additional process improves the overall diagnostic accuracy of our service, thus reducing error.

Some ED radiographs such as obvious dislocations or fractures, or injuries of fingers and toes may not require formal reporting by a radiologist. The adoption of a selective reporting policy may reduce the reporting workload of the radiology department without compromising patient care^4^ and may have financial incentives in the form of lesser reporting costs. It is important to note that in McConnell et al.'s study,^5^ the authors suggested that the accuracy of image interpretation may be higher when a radiographer's PIE is combined with an ED medical practitioner's interpretation.

Radiographer PIE is likely to benefit both the patients and ED in reducing missed injuries and reducing recall rates. For any ED who is planning on implementing such a system, it is important to clearly advertise and communicate the process prior to implementation with regular reminders of the availability of such a process. Regular audits on efficiency and a robust verification platform are crucial to the effective functioning of PIE by radiographers. Further research into comparing the accuracy of ED referrer interpretation and radiographer evaluation of radiographic images and also the combined accuracy of the ED referrer and radiographer versus radiologist's interpretation will give better understanding of the future direction of the radiographer image interpretation role to improve patient outcomes in ever‐demanding ED.

## Conflict of interest

The author declares no conflict of interest.
